# Characterization of the genomic sequence data around common cutworm resistance genes in soybean (*Glycine max*) using short- and long-read sequencing methods

**DOI:** 10.1016/j.dib.2020.106577

**Published:** 2020-12-09

**Authors:** Eri Ogiso-Tanaka, Nobuhiko Oki, Tsuyoshi Tanaka, Takehiko Shimizu, Masao Ishimoto, Makita Hajika, Akito Kaga

**Affiliations:** a Institute of Crop Science, National Agriculture and Food Research Organization (NARO), 2-1-2 Kannondai, Tsukuba, Ibaraki 305-8518, Japan; b Kyushu Okinawa Agricultural Research Center, NARO, 2421 Suya, Koushi, Kumamoto 861-1192, Japan

**Keywords:** *Glycine max*, Soybean, Resistance to the common cutworm, QTL region, Whole genome resequencing, Targeted amplicon sequencing, HiSeq, Oxford Nanopore MinION

## Abstract

The common cutworm (CCW, *Spodopteraab litura* Fabricius) is one of the pests that most severely infect soybean (*Glycine max* L. Merr.). In a previous report, quantitative trait loci (QTL) analysis of CCW resistance using a recombinant inbred line derived from a cross between a susceptible cultivar ‘Fukuyutaka’ and a resistant cultivar ‘Himeshirazu’, identified two antixenosis resistance QTLs, *CCW-1* and *CCW-2*. To reveal sequence variation between the aforementioned two cultivars, whole genome resequencing was performed using Illumina HiSeq2000 (75,632,747 and 91,540,849 reads). The generated datasets can be used for fine mapping and gene isolation of *CCW-1* and *CCW-2* as well as for revealing more detailed genetic differences between ‘Fukuyutaka’ and ’Himeshirazu’ .

## Specification Table

**Table:** 

Subject	Plant science
Specific subject area	Agricultural and Biological Sciences, Genomics of soybean (*Glycine max*)
Type of data	Figure and fastq/fasta files
How data were acquired	Whole genomes of soybean cultivars ‘Fukuyutaka’ and ‘Himeshirazu’ were sequenced using the ILLUMINA HiSeq2000 short-read sequencer. The sequence of the unique genomic region in *CCW2* was amplified by genomic polymerase chain reaction (PCR) and sequenced using MinION nanopore long-read sequencer (type R9.4, Oxford Nanopore Technologies Ltd., UK [ONT]).
Data format	Raw sequencing reads (fastq), Binary Alignment Map (BAM) and analyzed files (fasta)
Parameters for data collection	The common cutworm susceptible soybean cultivar ‘Fukuyutaka’ and resistant cultivar ‘Himeshirazu’ were used in this work. Their seeds are available from Genebank in NARO (https://www.gene.affrc.go.jp/databases_en.php). Genomic DNA for the sequencing was prepared from new leaves of one individual.
Description of data	HiSeq: Sequencing libraries were prepared with 1 μg DNA input, using the TruSeq DNA PCR-Free Library Preparation Kit (Illumina). Library pools were quantified by qPCR, loaded on the HiSeq2000 patterned flow cells and clustered on an Illumina cBot in accordance with the manufacturer's protocol. Flow cells were sequenced on the Illumina HiSeq2000 with 2 × 100 bp reads. Demultiplexing of sequencing data was performed with bcl2fastq2. MinION: Amplicons were obtained by amplification from the genomic DNA of ‘Himeshirazu’. A total of 1 µg amplicon was end-repaired and used for library construction. The MinION sequencing was run using MinKNOW (version 1.7.3). The resulting FAST5 files were converted to FASTQ files using the Albacore basecaller (version 1.1.0, ONT). The raw reads were assembled using Canu (version 1.6) [1].
Data source location	Institute of Crop Science, National Agriculture and Food Research Organization (NARO), Tsukuba, Japan
Data accessibility	The sequence data have been deposited in the DNA Data Bank of Japan Sequence Read Archive, under submission ID DRA010742, DRA010747, DRA010652 http://trace.ddbj.nig.ac.jp/DRASearch/ (BioSample accessions: PRJDB10367, PRJDB10313) The sequence has been placed in fasta format on FigShare, https://figshare.com/search?q=10.6084%2Fm9.figshare.13220792

## Value of the Data

• The genomic data of the susceptible and resistant soybean cultivars of common cutworm can be used for the development of a molecular marker for detecting quantitative trait loci and isolating genes.
• The sequence data for insert genomic region of ‘Himeshirazu’ in the *CCW2* region can be used for fine-mapping of a candidate gene.
• These data can be used for development of DNA markers and can contribute to marker-assisted selection in soybean breeding.

## 1 Data Description

The common cutworm (CCW, *Spodoptera litura* Fabricius) is one of the most serious pests of soybean (*Glycine max* (L.) Merr.). Komatsu et al (2004) reported on the antibiotic effects of soybean cultivars ‘Fukuyutaka’ and ‘Himeshirazu’ on CCW. ‘Fukuyutaka’ is a leading cultivar in southwestern Japan but is susceptible to CCW. ‘Himeshirazu’ is a forage cultivar but has strong CCW resistance [2]. Quantitative trait loci (QTL) analysis of CCW resistance using a recombinant inbred line derived from a cross between ‘Fukuyutaka’ and ‘Himeshirazu’, identified two antibiosis resistant QTLs, *CCW-1* and *CCW-2* [3], [4], and two antixenosis resistant QTLs, *qRslx1* and *qRslx2* [5]. The QTLs, *CCW-1* and *CCW-2*, regions of ‘Himeshirazu’ were verified by using near isogenic lines [6]. To detect the polymorphic sites of *CCW-1* and *CCW-2* genomic regions, we performed whole genome resequencing and variant detection.

**Fig. 1:**
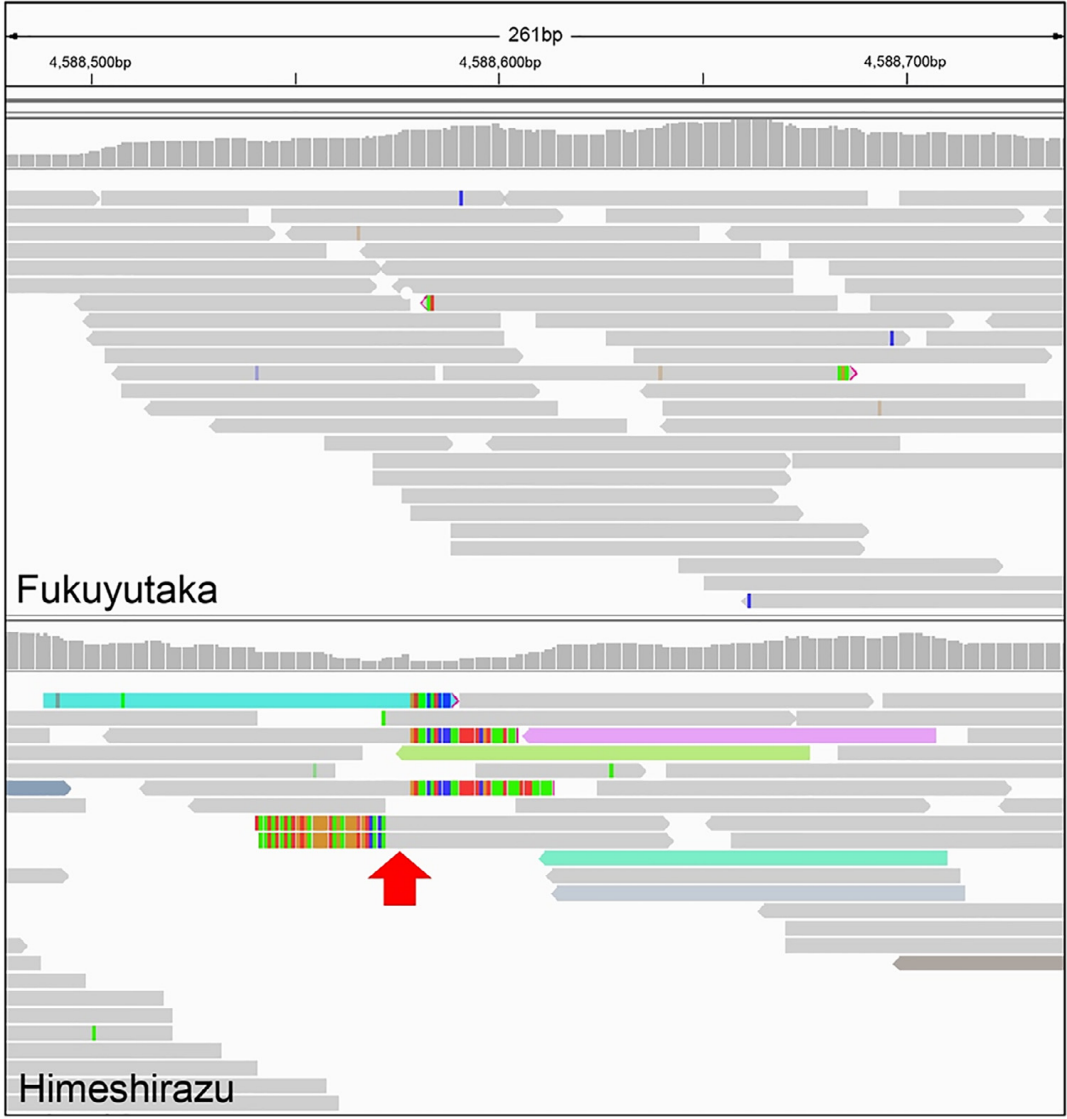
Read alignment suggests an insertion event around Chr7:4588573-4588578 in ‘Himeshirazu’ (bottom panel). The plot is an image from the integrative genome viewer that represents the read alignment of ‘Fukuyutaka’ (top panel) and ‘Himeshirazu’ (bottom panel). The red arrow represents the position where the insertion sequence is presumed to be located.

**Fig. 2:**
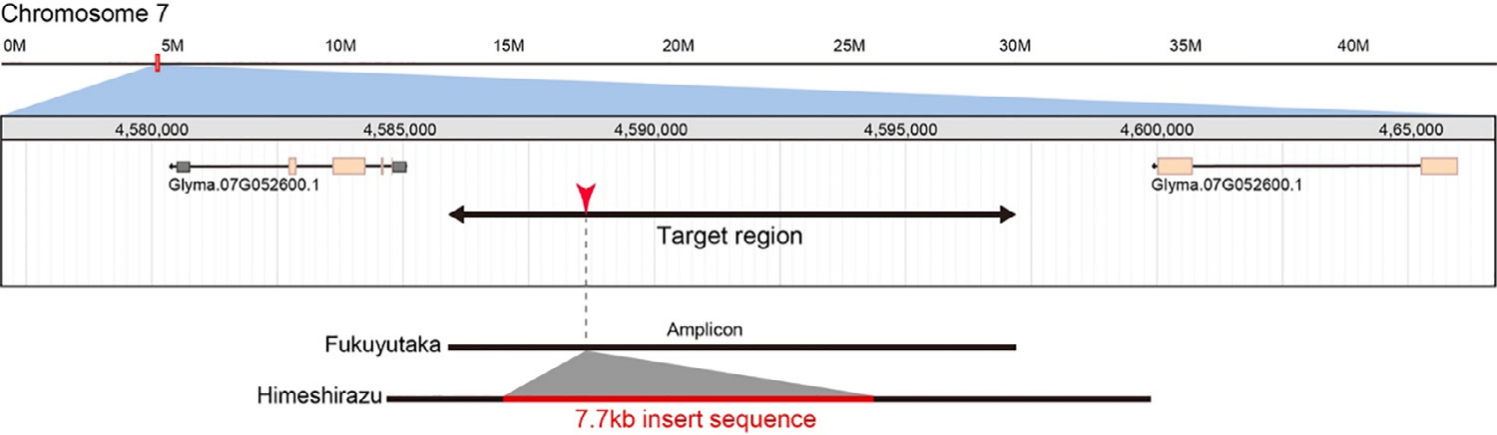
Position and size of the amplicon for targeted sequencing with MinION platform (Chr07:4585885-4597201, amplicon size=11 kbp in reference genome). Black and red arrows represent target region of amplicon sequencing and candidate insertion breakpoint, respectively. Black lines represent amplicons from ‘Fukuyutaka’ and ‘Himeshirazu’.

HiSeq: We present the whole genome sequence data of ‘Fukuyutaka’ and ‘Himeshirazu’. We sequenced paired-end libraries using the Illumina HiSeq2000 and generated 75,632,747 and 91,540,849 reads. These were compared to the reference genome version 2.0 (Gmax275: http://genome.jgi.doe.gov/pages/dynamicOrganismDownload.jsf?organism=Phytozome#, downloaded on May 15, 2015) [7], and 1,599,492 and 1,846,338 polymorphic sites were detected in ‘Fukuyutaka’ and ‘Himeshirazu’, respectively (Table 1). Among them, the number of polymorphic sites in the *CCW1* region (Chr7:10,655,942-15,394,281 corresponding to the genomic interval of SSR markers, Sat_258-Satt175) was 2,489 for ‘Fukuyutaka’ and 4,873 for ‘Himeshirazu’, and 9,553 and 10,627 polymorphisms were detected in the *CCW2* region (Chr7:4,559,713-8,283,465 to Satt567-Satt463) for ‘Fukuyutaka’ and ‘Himeshirazu’, respectively (Table 1). The number of polymorphic sites between ‘Fukuyutaka’ and ‘Himeshirazu’ was 2,899 (SNP: 2,483, InDel: 416) in the *CCW1* region and 10,547 (SNP: 8,632, InDel: 1,915) in the *CCW2* region, (Table 2, S1-2). These data will be useful to develop SNP/InDel markers for genetic mapping and identify the responsible genes and comparative functional genomics. In addition, we found partially unaligned reads in ‘Himeshirazu’ around Chr7:4,588,573-4,588,578 (Fig. 1) from the read alignment. We successfully amplified about a 18 kb bp fragment of ‘Himeshirazu’ using primers flanking this unaligned region based on the Gmax275 reference genome (Chr07:4,585,885-4,597,201, 11,316bp in reference genome), and characterized the sequence in-depth (Fig. 2).

**Table 1:** Number of polymorphic sites. Differences from the reference genome (cultivar: Williams 82).

	Fukuyutaka	Himeshirazu
Whole genome	1599492	1846338
*CCW1* region (Sat_258-Satt175)	2489	4873
*CCW2* region (Satt567-Satt463)	9553	10627

**Table 2:** Number of polymorphic sites. Differences between ‘Himeshirazu’ and ‘Fukuyutaka’ cultivars.

	*CCW1* region (Sat_258-Satt175)	*CCW2* region (Satt567-Satt463)
SNP	2483	8632
InDel	416	1915
Total	2899	10547

**Fig. 3:**
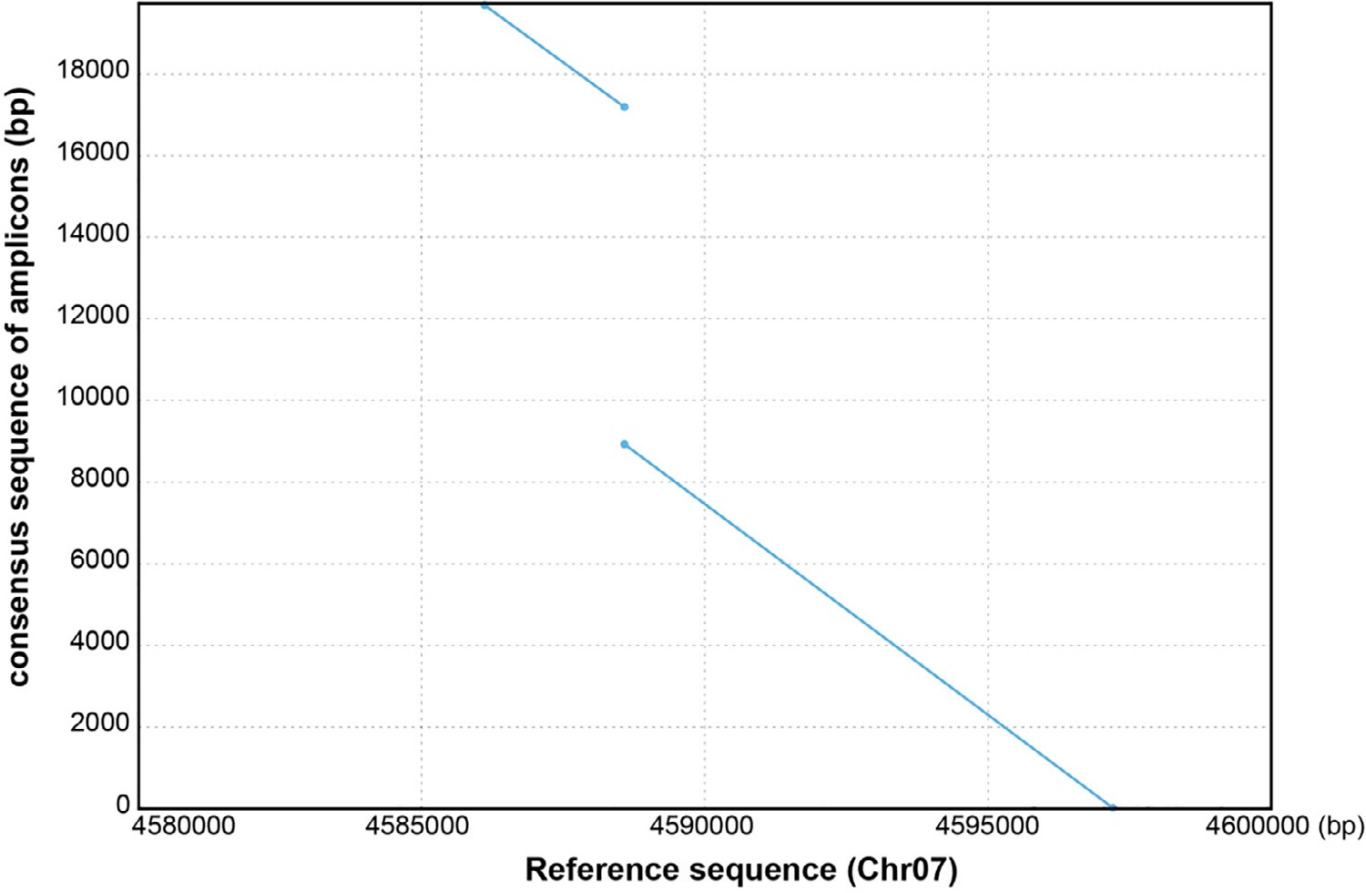
Genome alignment between consensus sequence of amplicons (y-axis) and the genomic sequence from 4.58 to 4.6 Mbp on Chr7 of Gmax275 reference genome (x-axis). The insertion breakpoint junction was on Chr07:4588576-4588579 (TGGA).

MinION: We determined the inserted sequences in the *CCW2* region observed in ‘Himeshirazu’. The amplified fragment, whose length was about 18 kbp estimated from PCR analysis, was sequenced using the Oxford Nanopore MinION platform (Oxford Nanopore Technologies Ltd., Oxford, UK). We obtained 28,725 raw reads. Only 18 reads were remained after the trimming and quality controls by Canu. The length distribution of 18 reads was bipolarized between 18,023 bp to 41,188 bp (Table 3). From the estimated size of the regions, we considered the longer reads would be artifacts. To confirm the possibility, we conducted homology search among 18 reads by BLASTN. While 14 shorter reads had one homologous region with each other, four longer reads (No. 15–18) whose lengths were 34,355 bp, 33,401 bp, 36,324 bp and 41,188 bp, respectively, had two homologous regions to short reads. We confirmed tandem duplication of a shorter read on a long read by mummer-4.0.0beta2 [8]. We also conducted a homology search of 18 reads against Gmax275 genome sequences and found the homology on Chr07 with gaps (7.2–7.5 Kbp) (Table 3). Therefore, we concluded that the longer reads were chimeric reads and excluded from the assembly. Finally, we constructed a consensus sequence from 14 reads. We also confirmed that the consensus sequence contained a target insertion observed in ‘Himeshirazu’ compared with the regions on Chr07 of the Gmax275 reference genome sequence with a long gap (Fig. 3). These data will be useful to perform fine mapping of *CCW-2* and identify the responsible gene.

**Table 3:** Summary of blastn results. The 18 “pass” reads aligned to target the sequence of the reference genome (Gmax275).

No.	Query length (bp)	Subject	Identity	Query start	Query end	Subject start	Subject end	Estimated Gap length(bp)
1	18023	Chr07	96.384	17	8350	4597201	4588579	
			95.64	15619	18002	4588582	4586108	7270
2	18100	Chr07	96.26	21	8367	4597201	4588573	
			94.353	15739	18097	4588582	4586106	7373
3	18118	Chr07	95.799	1	8310	4597173	4588573	
			96.739	15797	18118	4588582	4586192	7488
4	18149	Chr07	96.499	14	6546	4597201	4590470	
			96.884	6586	8298	4590365	4588602	
			96.734	15736	18146	4588582	4586106	7439
5	18150	Chr07	96.239	15	8360	4597201	4588573	
			97.264	15691	18117	4588582	4586100	7332
6	18163	Chr07	96.677	11	8387	4597201	4588573	
			96.802	15769	18163	4588582	4586113	7383
7	18172	Chr07	96.582	3	8330	4597166	4588573	
			96.529	15771	18170	4588582	4586108	7442
8	18185	Chr07	96.835	6	8384	4597188	4588573	
			97.029	15754	18184	4588582	4586093	7371
9	18212	Chr07	97.068	10	8370	4597201	4588613	
			95.858	15811	18212	4588582	4586100	7442
10	18216	Chr07	96.466	11	8336	4597168	4588573	
			96.879	15766	18211	4588582	4586090	7431
11	18217	Chr07	96.665	2	8382	4597196	4588573	
			97.457	15790	18216	4588582	4586111	7409
12	18222	Chr07	96.764	8	8375	4597197	4588586	
			97.57	15802	18222	4588582	4586117	7428
13	18269	Chr07	96.813	20	8396	4597196	4588573	
			96.121	15876	18268	4588582	4586111	7481
14	18272	Chr07	96.538	21	8401	4597201	4588573	
			97.065	15848	18110	4588582	4586269	7448
15	34355	Chr07	96.295	1	2609	4585889	4588582	
			96.922	10076	18488	4588573	4597202	7468
			90.363	18489	21065	4585885	4588582	
			94.983	28504	34355	4588573	4594619	7440
16	33401	Chr07	95.634	1	2579	4585907	4588582	
			95.221	10000	17555	4588573	4596411	7422
			91.039	17544	20127	4585885	4588582	
			94.494	27522	33401	4588573	4594679	7396
17	36324	Chr07	94.271	1	8259	4597196	4588573	
			90.693	15535	18055	4588582	4585914	7277
			94.253	18074	26331	4597205	4588573	
			97.425	33710	36324	4588582	4585904	7380
18	41188	Chr07	97.039	1	8374	4597179	4588573	
			96.272	15836	18461	4588582	4585885	7463
			91.788	18462	20978	4597202	4594636	
			88.625	20974	23209	4594839	4597202	
			90.557	23214	25741	4585889	4588582	
			95.204	32940	41187	4588573	4597196	7200

No: Number of queries (“pass” reads determined using MinION)

Identity: Percentage of identity (identical site/denominator).

Query start - Q uery end: query range coved by alignment

Subject start - Subject end: subject range covered by alignment.

Estimated Gap length (bp): The subject length and physical position on

## 2 Experimental Design, Materials and Methods

### 2.1 Sample collection and DNA extraction

Samples for HiSeq: Soybean cultivars ‘Fukuyutaka’ and ‘Himeshirazu’ were cultivated in a greenhouse at the National Agriculture and Food Research Organization (NARO) in Tsukuba, Ibaraki, Japan, and treated in dark condition for one-week to reduce organelle before DNA extraction. Leaves were collected from about five seedlings of ‘Fukuyutaka’ and ‘Himeshirazu’ (seeds from a single individual), and DNA was extracted from bulked leaves using a protocol from Peterson et al. [9] with some modification.

Samples for MinION: ‘Himeshirazu’ was cultivated in an artificial climate chamber at NARO. Genomic DNA was extracted from the newest fresh leaves of ‘Himeshirazu’ using the CTAB method with the following modifications: Leaves were homogenized in liquid nitrogen and the tissues were transferred to preheated 2 x CTAB DNA extraction buffer (2% CTAB, 0.1 M Tris-HCl pH 8.0, 1.4 M NaCl, 1% PVP, 20 mM EDTA) and 80 μg/ml proteinase K. Then, they were incubated in a water bath at 55 °C for 15 min, and mixed occasionally by gentle inversion of the tubes. After they were removed from the water bath and the same volume of chloroform-isoamylalcohol (24:1) was added, they were mixed by inversion. They were spun down at 3000 rpm and the supernatant was transferred to the new tube. Equal volume of supernatant was added to isopropanol. They were mixed by inversion and centrifuged at 14000 rpm for 5 min (MX-201, TOMY Seiko Co., Ltd, Tokyo, Japan). The pellets were washed with 70% ethanol twice and dried at room temperature. The DNA pellet was air-dried and dissolved in 50 μl of low TE buffer (10 mM Tris-HCl, 0.1 mM EDTA pH 8.0). The DNA concentration was measured by nanodrop (Thermo Fisher Scientific Inc., USA) and Qubit (Thermo Fisher Scientific Inc.).

### 2.2 Library preparation, illumina and nanopore sequencing

HiSeq: A total of 75632747 and 91540849 paired reads from ‘Fukuyutaka’ and ‘Himeshirazu’ of a 350-bp insert-size library by TruSeq DNA PCR Free kit (Illumina Inc., San Diego, CA, USA) were generated from the Illumina HiSeq2000. The reads derived from the HiSeq2000 sequencing data were processed to remove adapter sequences and low-quality bases by trimmomatic-0.30 using the option “ILLUMINACLIP:adapter.fa:2:30:10 LEADING:15 TRAILING:15 SLIDINGWINDOW:4:15 MINLEN:32” [10]. The FASTQ files after quality control were deposited in the Sequence Read Archive (SRA) (biosample accession number: SAMD00238602 and SAMD00238603) under the bioproject accession number DRA010742 (Fukuyutaka) and DRA010747 (Himeshirazu).

MinION: 10 ng DNA from ‘Himeshirazu’ were used in the PCR reaction with primers CCW2-2_F (5’-TGACTGATCCTGCTGTGAGAATGTT-3’) [Chr07:4559602-4559619] and CCW2-8_R (5’-TGTAACGTAGGAAAATGACAACACTACATC-3’) [Chr07:4602994-4602971] for the amplification of approximately an 11-kb region in the reference Gmax275 genome. PCR was performed using the GeneAmp PCR PCR System 9700 (Thermo Fisher Scientific Inc.) using PrimeSTAR GXL DNA Polymerase (Takara Bio Inc., Shiga, Japan). The PCR conditions were as follows: initial denaturation at 94 °C for 1 min, 30 cycles of denaturation at 98 °C for 10 s, and annealing and extension at 68 °C for 10 min. The PCR products were electrophoresed on 0.8% agarose gel using the HindIII DNA ladder (Takara Bio Inc., Shiga, Japan) and stained with ethidium bromide. The amplicon size from ‘Himeshirazu’ was approximately 18 kb (between 9416 bp and 23130 bp fragment of HindIII marker). The amplicon (1 µg) was end-repaired and dA-tailed using the NEBNext End-Repair and NEBNext dA-Tailing modules (New England Biolabs, MA, USA) according to the manufacturer's instructions. Then, the sequencing adapter was ligated to the dA-tailed DNA using the Blunt/TA Ligase Master Mix (New England Biolabs, MA, USA) according to the manufacturer's instructions using the 1D Amplicon Sequencing SQK-LSK108, R9 version (Oxford Nanopore Technologies Ltd.). Sequencing was performed using a MinION flow cell (R9.4, ONT) in the MinION portable sequencer (Oxford Nanopore Technologies Ltd.). The sequencing run was performed using the MinKNOW software (version 1.7.3, Oxford Nanopore Technologies Ltd.) with the live basecalling option disabled. The run time was 31 h. The resulting FAST5 files in the “pass” folders, which correspond to sequences with high quality scores, were converted to FASTQ files using the Albacore basecaller (version 1.1.0, ONT). The FASTQ file was deposited in the SRA (biosample accession number: SAMD00238644) under the bioproject accession number DRA010652. The analyzed sequence file (FASTA) was deposited to figshare (doi:10.6084/m9.figshare.13220792).

### 2.3 Variant call and coverage analysis using HiSeq short-read sequence data

After trimmed paired reads were mapped on the soybean genome reference (Gmax275: http://genome.jgi.doe.gov/pages/dynamicOrganismDownload.jsf?organism=Phytozome#, downloaded on May 15, 2015) [7] using BWA-MEM [11]. We obtained mapping rates of 99.4% and 99.2% with 94.2% and 95.8% coverage of the reference (with x13.5 and x13.3 coverage of the covered regions) from ‘Fukuyutaka’ and ‘Himeshirazu’. Reads were then preprocessed using samtools v.1.3.1 [12] to convert SAM into BAM, which was sorted by coordinate order. Duplicate reads were marked using Picard MarkDuplicates (v.2.7.1) with the option “ASSUME_SORTED=true REMOVE_DUPLICATES=true” (http://broadinstitute.github.io/picard/). For local realignment and base quality score recalibration of the mapped reads, the tools RealignerTargetCreator, IndelRealigner, and BaseRecalibrator from GATK (Genome Analysis Toolkit) v.3.7.0 [13] were applied. All tools were used with the recommended standard settings [14,15]. This workflow design is in accordance with the best practices from the Broad Institute. Variants were called using the tool HaplotypeCaller with the option “–emitRefConfidence GVCF -variant_index_type LINEAR -variant_index_parameter 128000.” They were filtered with the filtering option “DP>100 || DP<5 || QD < 2.0 || FS > 60.0 || MQ < 40.0 || MQRankSum < -12.5 || ReadPosRankSum < -8.0” by VariantFiltration from GATK (McKenna et al., 2010). Then we generated a combined GVCF file with dbSNP using GenotyepGVCFs from GATK (McKenna et al., 2010). The dbSNP file was downloaded from NCBI (downloaded on May 31, 2016, from the dbSNP site of NCBI, which is now closed; currently, dbSNP information on soybean is being accessed from the European Variation Archive at EMBL-EBI). Because the reference genome (Glycine max v2.0) listed in NCBI and Gmax275 in Phytozome have different physical positions in some sequences, we created and used a modified dbSNP file for the Gmax275 position.

### 2.4 Identification of the unique genomic sequence in the *CCW-2* region of ‘Himeshirazu’ using MinION long-read sequence data

The 28,725 reads derived from the MinION sequencing platform were input to canu-1.6 with the options (-p asm -d gmax_amplicon genomeSize=15000 correctedErrorRate=0.5 -nanopore-raw all.fastq gnuplotTested=true useGrid=false). After quality control and trimming, only 18 long reads were remained. The homologies of the 18 reads to *CCW-2* regions were analyzed by blastn in the BLAST+ [16] and detected an insertion region of 7.2–7.5kb that did not hit the reference sequence (Table 3). Four of 18 reads showed tandem repeat sequence, and the length of the read was about twice the size of the PCR product, suggesting that the four reads are a chimera. Then, by using 14 MinION reads, a consensus sequence was generated. From the consensus sequence, 7.7kb insertion (breakpoint junction on Chr07:4588576-4588579 [TGGA]) was detected by comparing with Gmax275 reference genome (Fig. 3).

## Declaration of Competing Interest

The authors declare that they have no competing financial interests or personal relationships that can influence the work reported in this paper.

## Appendix Supplementary materials

Image, application 1

## Footnotes

Supplementary material associated with this article can be found in the online version at doi:10.1016/j.dib.2020.106577.
